# Wrestling the Two-Headed Hydra: On the Consequences of the Bifurcated Concept of “Undertaking” in EU Competition Law

**DOI:** 10.1017/jme.2025.10139

**Published:** 2025

**Authors:** Łukasz Grzejdziak

**Affiliations:** Law, University of Strathclyde, Glasgow, United Kingdom; Sutherland School of Law, University College Dublin, Dublin, Ireland; Faculty of Law and Administration, University of Lodz, Lodz, Poland

**Keywords:** EU Competition Law, EU Social Law, Concept of Undertaking, Healthcare and Economic Activity, EU State Aid Law, the Notion of State Aid

## Abstract

The evolution of the CJEU’s jurisprudence has led to the emergence of a distinct, sector-specific notion of economic activity in the context of services delivered within public healthcare systems. This interpretation diverges markedly from the general framework applied in other sectors. This form of conceptual dualism lacks a clear normative foundation in the provisions of the TFEU and poses a potential challenge to the integrity of the role assigned to services of general economic interest under both the Treaty and established CJEU case law. Significantly, the exclusion of practically all activities within public healthcare systems from the ambit of EU competition law has the potential to generate significant distortions of competition. This is particularly relevant in the context of healthcare systems, such as that of Poland, which exhibit a mixed structure and where public and private providers engage in substantial competition.

## Introduction

The application of EU competition law to the healthcare sector has long been a matter of legal and policy controversy. This is due not only to the sector’s foundational role in meeting essential human needs but also to the complex intersection of competences between the Member States and the European Union, as well as the coexistence of principles of social solidarity and market-based competition. Although responsibility for healthcare policy and organization lies primarily with the Member States, the Union holds exclusive competence to establish competition rules necessary for the functioning of the internal market.

In recent years, growing pressure on national healthcare systems — arising from, inter alia, adverse demographic trends — has led Member States to increasingly incorporate competition mechanisms in order to enhance the efficiency of healthcare delivery. The tension between these competing values has prompted considerable academic debate, particularly concerning how best to delineate the boundary between national autonomy and EU-level market regulation.

This tension has also surfaced in the case law of the Court of Justice of the European Union (CJEU), especially in relation to the interpretation of the concepts of “undertaking” and “economic activity.” These concepts are jurisdictional cornerstones of EU competition law, including its provisions on state aid.^1^ Specifically: (1) only undertakings, as defined under EU law, may be subject to antitrust prohibitions; (2) concentrations between undertakings are subject to merger control; and (3) state aid rules apply only where selective economic advantages are granted to undertakings. Thus, the definitions of “undertaking” and “economic activity” directly shape the personal and material scope of competition law in the healthcare sector.

In this paper, I argue that the development of the CJEU’s case law has given rise to a distinct, sector-specific concept of economic activity applicable to services provided within public healthcare systems. This concept differs significantly from the general approach adopted in other sectors and is irreconcilable with it. Such a conceptual dualism finds no clear basis in the provisions of the Treaty on the Functioning of the European Union (TFEU), and it risks undermining the role of services of general economic interest (SGEIs) as envisaged in the Treaty and earlier CJEU jurisprudence. SGEIs are commercial services of general economic utility subject to public-service obligations. According to TFEU Article 106(2), undertakings entrusted with the operation of SGEIs enjoy a derogation from the rules on competition, including state aid law and the rules addressed to undertakings, insofar as the application of such rules does not obstruct the performance, in law or in fact, of the particular tasks assigned to them. The current approach significantly limits the scope of this derogation in relation to healthcare services.

More fundamentally, the de facto exclusion of all public healthcare system activities from the scope of EU competition law may result in significant distortions of competition. I demonstrate the practical implications of this phenomenon through an analysis of the Polish healthcare system.

The article proceeds as follows. First, I trace the evolution of the CJEU’s jurisprudence on the classification of healthcare-related activities as economic or noneconomic. This analysis is structured around three distinct phases of development. Subsequently, I examine the key organizational features of the Polish healthcare system and assess the consequences that the non-application of EU competition rules has on its operation and efficiency.

## The General (Abstract) Concept of Undertaking and Economic Activity Under EU Rules on Competition

In the early years of case law development, the CJEU occasionally applied the institutional (subjective) notion of an undertaking understood as “an undertaking is constituted by a single organization of personal, tangible and intangible elements, attached to an autonomous legal entity and pursuing a given long term economic aim.”^2^ However, the CJEU soon definitively adopted a position on the functional nature of this concept, focused on the tasks performed by a given entity.

Such a criterion was applied in the landmark judgment in the case of *Höfner and Elser v. Macrotron*,^3^ where the German federal employment agency was considered an undertaking. In this ruling, the CJEU held that an “undertaking encompasses every entity engaged in an economic activity, regardless of the legal status of the entity and the way in which it is financed and, secondly, that employment procurement is an economic activity.”^4^ This ruling effectively detached this concept from the subjective (institutional) element, allowing several natural or legal persons or even part of the activities of a given natural or legal person to be classified as an undertaking.

The concept of economic activity has been interpreted inconsistently. In his opinion in the *FENIN* case, Advocate General Poiares Maduro aptly distinguished the two criteria used by the CJEU to define economic activity and thus the concept of undertaking, comparative, and market.^5^

The first criterion applied in the landmark case of *Hofner and Elser v Macrotron* boils down to establishing the possibility for certain activities to be carried out not only by public but also by private entities.^6^ The possibility of conducting similar activities by a private entity does not seem to be the most accurate. It does not precisely explain how to deal with a situation in which similar activities are carried out by private and public entities, but on different conditions.

The market criterion seems more useful. It focuses on the existence of a market for specific goods or services, or competition in offering specific goods or services in the market. In this context, as held by the CJEU in the case of *Italy v Commission*, an entity “exercises an economic activity inasmuch as it offers goods and services on the market.”^7^

As Advocate General Maduro noted, “It is not the mere fact that the activity may, in theory, be carried on by private operators which is decisive, but the fact that the activity is carried on under market conditions.”^8^ Accordingly, any activity should be considered economic if the resulting goods or services are subject to market exchange. This, in turn, may take place only if there is a potential to receive remuneration exceeding the cost of production of goods or offering services. Importantly, this does not exclude nonprofit entities from being classified as undertakings if they engage in such market-based activities.^9^

Arguably, the market criterion has been applied in the majority of cases,^10^ including *Casa di Risparmio de Firenze*, where the CJEU held, while assessing the activity of banking foundations, that “the banking foundation must be regarded as an undertaking, in that it engages in an economic activity, notwithstanding the fact that the offer of goods or services is made without profit motive since that offer will be in competition with that of profit-making operators.”^11^

The application of the market criterion implies that the concept of “economic activity” is defined in objective and substantive terms. Consequently, the classification of an entity as an undertaking does not depend on its affiliation with a particular sector or the purpose of its activity, but rather on the nature of the activity itself.

Moreover, the market test is based on a simple and logical assumption, both practically important and theoretically elegant. It should be remembered that the concept of economic activity determines the scope of the application of EU competition rules. The reliance on a competition-based criterion implies that the relevant rules may be applied in any context where competition is present and, consequently, warrants protection. *A contrario*, when there is no market for certain goods and services, there is no need to apply competition law.

This reasoning encompasses, inter alia, the exclusion from the scope of economic activity of those tasks “which fall within the exercise of public powers”^12^ or, as articulated by the CJEU in the *Cali* ruling, constitute “a task in the public interest which forms part of the essential functions of the State.”^13^ Therefore, as the Commission underlined in the Notice on the Notion of State Aid, the concept of economic activity does not include, inter alia, activities in the field of public administration, including the army or the police; air navigation safety and control; maritime traffic control and safety; anti-pollution surveillance; the organization, financing and enforcement of prison sentences; the development and revitalization of public land by public authorities; or the collection of data to be used for public purposes on the basis of a statutory obligation imposed on the undertakings concerned to disclose such data.^14^

The classification of such tasks as noneconomic is not altered by the fact that they are carried out in exchange for payment. The essential functions of the state are inherently noneconomic, as they do not involve the offering of goods or services on a market. Furthermore, where an activity is exclusively reserved for the state, the conditions necessary for the existence of a competitive market are, by definition, absent.

A fundamental limitation of the market criterion, evident from its inception, lies in its inability to provide a clear and consistent framework for classifying mixed activities — that is, activities that involve both the performance of essential state functions and which are, to some extent, carried out under competitive conditions.

On the one hand, it could be argued that since the existence of competition is a defining feature of market activity, even a limited degree of competition between operators should suffice to confer an economic character on the activity in question. From this perspective, excluding such activities from the scope of competition law would risk undermining the relevance of the competitive element itself. Indeed, competition between operators may be easily eliminated through coordination agreements or distorted through state intervention, such as the granting of aid. Such activity may nonetheless benefit from derogation from the Treaty provisions, including the rules on competition, under TFEU Article 106(2), provided that the exercise of essential state functions encompasses the provision of services of general interest and the services are of a market nature.

On the other hand, the mere presence of competitive elements does not necessarily imply that the underlying exchange of goods or services is market-based. Such elements can have only marginal importance.

The CJEU has addressed this issue in a number of judgments concerning activities marked by elements of social solidarity, suggesting that not all forms of competition entail an economic character within the meaning of EU law. The evolution of this line of case law can be analytically divided into three distinct phases. They are analyzed below.

## Activities Based on the Principle of Social Solidarity and Economic Activity

### Phase 1 — The Establishment of the Sector-Specific Test

In *Poucet*, the CJEU held that activities carried out within the framework of a compulsory pension, health, and maternity insurance scheme for self-employed persons had noneconomic character.^15^ The Court emphasized that the scheme was mandatory, accessible to all participants irrespective of income level, and based on the principle of solidarity, as evidenced by contributions being calculated in proportion to the insured’s income.^16^

Consequently, the CJEU concluded that “Sickness funds, and the organizations involved in the management of the public social security system, fulfil an exclusively social function. That activity is based on the principle of national solidarity and is entirely non-profit-making. The benefits paid are statutory benefits bearing no relation to the amount of the contributions.”^17^

The CJEU’s reasoning in *Poucet* was, at the time, subject to criticism for its apparent inconsistency with the functional concept of economic activity.^18^ Although the Court referred expressly to the *Macrotron* ruling, it did not apply a strictly functional test. Instead, it took into account social considerations related to the fund’s purpose and the method of its financing.^19^ This approach was rightly interpreted as a move toward delineating a sphere of Member State autonomy that falls outside the scope of EU competition law.^20^ Moreover, it contributed to the recognition of solidarity as a fundamental principle of European law.^21^ By doing so, the Court effectively allowed Member States to maintain a certain degree of discretion in the organization and implementation of their social policy objectives.

The line of case law developed in *Poucet* was continued by the CJEU in *Cisal*, where considerations of basing the activity on the principle of social solidarity were decisive in the Court’s finding that the Italian compulsory insurance fund for accidents at work and occupational diseases did not constitute an undertaking within the meaning of the EU competition law.^22^ Again, the CJEU relied on arguments about the essentially social objective of the activity^23^ and the lack of a direct link between the amount of the contributions paid and the benefits obtained by the insured, which in turn demonstrated the existence of solidarity between the insured.^24^

This approach was reaffirmed in subsequent CJEU cases of *FFSA*,^25^ *Albany*,^26^ and *Brentjens.* ^27^ Here, the CJEU, applying the sector-specific test, concluded that the activities of entities managing insurance funds could, under certain conditions, be classified as economic in nature. In *FFSA*, the fund in question pursued a social, nonprofit objective and exhibited some elements of social solidarity. However, these elements were not predominant.^28^ Participation in the scheme was voluntary, it operated on a capitalization basis, and the level of benefits received by insured persons depended solely on the amount of their contributions and the insurer’s financial performance. The Court emphasized that the fund carried on economic activity in competition with life assurance companies.^29^

Similarly, in *Albany*, the CJEU found that an entity managing a supplementary pension scheme established through a collective agreement qualified as an undertaking since it engaged in an economic activity in competition with insurance companies.^30^ The Court acknowledged that while elements of social solidarity may diminish a fund’s competitiveness, such features could nevertheless justify the granting of exclusive rights by a Member State in compliance with TFEU Article 106(2).^31^

These cases consolidated the foundations of the economic activity test as applied to activities of the bodies active in the framework of social security systems organized under the principle of social solidarity, such as entities managing the public health insurance systems and sickness funds. The emerging case law gave rise to what may be described as a sector-specific or, as J.W. van der Gronden named it, the “concrete” economic activity test.^32^ The test focuses on the features of a system under scrutiny as designed by national legislation. Where a system is predominantly structured around the principles of social solidarity — manifested, inter alia, in the pursuit of the public interest, the provision of services either free of charge or for a nominal fee, and the mandatory nature of contributions — entities responsible for managing such a system are not regarded as undertakings within the meaning of EU competition law. These criteria differ fundamentally from the objective and functional indicators employed under the general test, or what J.W. van de Gronden refers to as the “abstract” test.^33^

This test was developed alongside the generally applied market-based or comparative test, operating in parallel rather than as a replacement. Thus, differences between the existing tests made earlier criticisms of the *Poucet* judgment at least somewhat justified. However, the apparent divergence between the two approaches was not irreconcilable. It could be assumed that activities organized entirely under the social solidarity logic were governed by a fundamentally different rationale than market-based activities subject to competitive dynamics. Entities engaged in the former did not compete with one another, nor with market operators performing similar services on regular commercial conditions. Consequently, the application of the general test would likely yield the same result — namely, the classification of these activities as noneconomic. From this perspective, the sector-specific test may be viewed not as a departure from the general test, but rather as its contextualized variant tailored to the unique characteristics of certain types of activities.

The first phase of the development of the CJEU case law, therefore, led to the creation of a sectoral test for economic activity based on the nuanced approach to distinguishing between economic and noneconomic activities in contexts where social objectives intersect with market dynamics. They also highlight the relevance of both the degree of competition and the structural characteristics of a given scheme in determining its legal classification under EU competition law.

Social security systems of a mixed character — combining elements of both social solidarity and elements of competition — were classified as undertakings, even where their objectives were primarily social and not profit-driven. As illustrated by the *Albany* judgment, the CJEU’s case law has evolved to recognize three distinct standards of assessment in this context.

First, entities whose operations were predominantly organized around the principle of social solidarity were not regarded as engaging in economic activity and, consequently, fell outside the scope of EU competition rules. Entities whose activities incorporated significant, though not dominant, elements of social solidarity and operating in a competitive environment were considered undertakings. However, those of them entrusted with the operation of SGEI could benefit from the derogation under TFEU Article 106(2). The third category, consisting of the remaining undertakings, was subject to the full application of competition law. This tripartite framework reflected the Court’s attempt to balance social policy objectives with the internal market’s requirements, acknowledging the complexity of activities situated at the intersection of public interest and market-based service provision.

### Phase 2 — Towards the Social Exclusion from EU Competition Law

The judgment in *AOK Bundesverband* raised important questions regarding the consistency between the sector-specific and general tests for identifying economic activity.^34^ In this case, the CJEU held that the activities of German sickness insurance funds did not constitute economic activity, as they were carried out within the framework of the statutory health insurance system, which was based on the principles of social solidarity and served an exclusively social objective. The Court placed particular emphasis on the nonprofit nature of the funds and the absence of any direct correlation between the contributions paid and the benefits received.^35^

However, the German universal health insurance system diverged in important respects from those assessed in earlier cases. Notably, German insurance funds exercised a degree of discretion in setting contribution levels, and the system itself incorporated competitive elements by allowing funds to compete for insured individuals, intending to enhance overall efficiency. The CJEU noted, however, that pursuit of that objective did not in any way change the nature of the sickness funds’ activity.^36^ Finally, it concluded that the funds were “not in competition with one another or with private institutions as regards grant of the obligatory statutory benefits in respect of treatment or medicinal products which constitutes their main function.”^37^

This ruling illustrates the Court’s continued prioritization of the principle of solidarity and the social purpose of public insurance systems over the market or competition element, thereby reaffirming the sectoral approach to the classification of economic activity in the field of social and health security.

The *AOK Bundesverband* ruling has been criticized for its apparent inconsistency with the earlier CJEU’s case law.^38^ Advocate General Jacobs, in his opinion in the case, argued that the German health insurance funds did, in fact, engage in competition for the insured, particularly given the funds’ latitude in setting contribution values.^39^ He applied both comparative and market criteria tests and came to the conclusion that German *Krankenkasse* were undertakings since they competed with each other and with their private counterparts. At the same time, similar activity was pursued by private entities.

Indeed, the intensity of competition between German funds exceeded that observed in cases such as *Brentjens*, where the activities of the entities concerned were deemed economic in nature. Applying the general test — based on the existence of market activity and competition — to the facts of *AOK Bundesverband* would likely have led to a different conclusion.^40^

As such, the ruling represents a significant departure from the application of a uniform and objective definition of undertaking based on market or competition criteria. Instead, it illustrates the development of two parallel approaches: the general test and the sector-specific test. The latter incorporates additional considerations such as the nature and purpose of the activity and the degree of regulatory oversight involved.^41^ This shift could be interpreted as a return to an institutional or subjective notion of undertaking.

Moreover, the *AOK Bundesverband* judgment may be viewed as a step towards the establishment of a “social exclusion” from the EU competition law, as noted by M. Krajewski and M. Farley.^42^ In this respect, the ruling reflects a broader judicial effort to carve out a space for national social policy autonomy within the internal market, particularly in domains characterized by solidarity-based structures.

In *AOK Bundesverband* and earlier case law, the CJEU applied the sector-specific test to assess the activities of sickness insurance funds. In the *FENIN* judgment, issued a year before AOK Bundesverband, the Court of First Instance (CFI)^43^ extended the application of the sector-specific test to the purchasing activities of public entities managing hospitals within the Spanish public healthcare system, which operated downstream of the insurance funds’ activity.^44^

The Court found that these entities, although involved in the acquisition of goods (specifically, medicines), were not acting as undertakings within the meaning of EU competition law. This conclusion was based on the observation that their purchasing activities were inseparable from the provision of healthcare services carried out in the context of a system based on the principle of solidarity and universal coverage. Thus, the CFI articulated the so-called “upstream/downstream” principle, according to which the classification of an entity as an undertaking is not determined solely by the economic nature of a specific activity — such as the purchase or sale of goods — but rather by the broader context and purpose of the activity to which it is functionally linked.^45^

The *FENIN* judgment, confirmed on appeal by the CJEU,^46^ thus reinforced the logic of the sector-specific (concrete) test by confirming that the economic or noneconomic character of an activity must be assessed in its broader institutional and functional context, particularly in sectors where the principles of universality, solidarity, and nonprofit orientation prevail.

The *FENIN* ruling has been seen as establishing a de facto exclusion from the competition rules of the entire sector of medical services provided within the framework of a system based on the principles of social solidarity, that is, to the extent that the sectoral test established in the *Poucet* judgment was met.^47^ However, neither the CFI nor the European Court of Justice (ECJ), which affirmed the CFI judgment on appeal, referred expressly to the nature of the medical services provided in the framework of the Spanish system. The CFI analysis was limited to the assessment of transactions for the purchase or sale of goods for the purposes of this activity.^48^ The specific nature of the Spanish medical services sector, which was undoubtedly organized on the social solidarity principle and financed directly by the state, was also considered.

Following the *FENIN* judgment, uncertainties emerged regarding the scope of the approach established therein — specifically, whether the sector-specific test should apply to all healthcare service providers operating within sickness insurance systems based on the principle of social solidarity. Such an interpretation would entail the exclusion of not only the entities administering these systems but also healthcare service providers from the scope of EU competition rules. It is important to note that, according to established CJEU case law, healthcare service providers have generally been regarded as undertakings within the meaning of EU competition law.^49^ In *FENIN*, the Court’s assessment of the economic nature of the relevant activity was confined to a narrowly defined area, namely the procurement activities undertaken by managers of hospitals operating within taxation-funded healthcare systems — systems which inherently exhibit fewer market-based characteristics. As such, the *FENIN* ruling could not be readily extended to other dimensions of healthcare provision, particularly those situated within insurance-based systems, where competitive elements are more pronounced.^50^

Based on the *FENIN* ruling, the Commission in its Notice on the Notion of State Aid expressly mentioned that public hospitals, which in some Member States are an integral part of a national health service and are almost entirely based on the principle of solidarity, are directly funded from social security contributions and other state resources, and provide their services free of charge on the basis of universal coverage, are not undertakings within the meaning of the EU competition law. The Commission distinguished such entities from hospitals and other health care providers that offer their services for remuneration, be it directly from patients or from their insurance. As the Commission underlined, in such systems, there is a certain degree of competition between hospitals concerning the provision of health care services, and thus, the fact that a health service is provided by such a public hospital is not sufficient for the activity to be classified as noneconomic.^51^

This approach reflects a relatively narrow interpretation of the *FENIN* judgment, whereby the classification of activities as noneconomic is largely confined to hospitals operating within taxation-funded healthcare systems. The position adopted by the European Commission in its Notice subsequently served as a point of reference for the Polish Competition Authority (UOKiK), which also holds responsibilities in the area of state aid monitoring. Drawing upon this interpretation, the Polish National Competition Authority (NCA) published on its official website a set of guidelines concerning the application of state aid rules to healthcare providers operating within the Polish system:

“The characteristics of the organization of public health care in Poland indicate that this system operates based on the principle of solidarity. Consequently, this means that medical entities, when providing medical services financed from public funds to citizens covered by compulsory health insurance, and therefore within the public system, do not act as undertakings. For this reason, it should be considered that support provided in connection with the provision of medical services within the public health care system is not subject to the provisions on State aid.”^52^

Furthermore, the Polish NCA noted that, even if healthcare providers operating within the public system were to be considered undertakings, public subsidies granted to such entities would likely still fall outside the scope of state aid rules. This is because the conditions relating to the distortion or threat of distortion of competition and the effect on trade between Member States would not be fulfilled. According to the NCA, “services financed by the National Health Fund are, in principle, addressed to Polish citizens and are provided within a system established to implement the state’s responsibilities in the field of public health protection.”^53^ Consequently, it is generally presumed that the provisions on state aid do not apply to entities operating within the public healthcare system. In line with the principle of separation, the NCA also recalled that entities providing both publicly financed and commercially offered healthcare services must either fully separate these two types of activities institutionally or maintain separate accounting records for each.^54^

In this manner, the Polish NCA made explicit its interpretation that, in light of the *FENIN* judgment, all activities conducted within the Polish public healthcare system are noneconomic and therefore fall outside the scope of EU competition law. This interpretation has been the subject of considerable controversy, as the Polish healthcare system differs substantially from the Spanish model examined in *FENIN.* In particular, the Polish system is insurance-based rather than taxation-funded and combines elements of social solidarity with market mechanisms. The structure of the Polish healthcare system will be discussed in detail in the section “The Consequences of the Application of the Sector-Specific Test to the Polish Healthcare System” below, where I will also address the implications of excluding entities operating within this system from the application of EU competition rules.

In conclusion, the second phase marked two significant developments in the evolution of the CJEU’s case law. First, it broadened the application of the sector-specific test to include activities organized based on both market mechanisms (i.e., competition) and the principle of social solidarity. To identify an economic activity, it was no longer sufficient to demonstrate the mere existence of competition between specific entities. Instead, it became necessary to balance the presence of market-oriented and solidarity-based elements and to assess which of these predominated. The judgment in *AOK Bundesverband* did not provide a definitive criterion for determining the threshold at which market elements would outweigh social considerations, such that the activity would be classified as economic. It was, however, evident that the presence of solidarity-based features was accorded greater weight in this evaluative process. This shift signified that the sector-specific test was no longer a variant of the general undertaking test, but rather a distinct and incompatible method of determining the existence of an economic activity. The second development concerned the extension of the sector-specific test to entities delivering healthcare services within systems organized according to the principle of social solidarity.

### Phase 3 — The Establishment of the Social Solidarity Exclusion from EU Competition Law

#### The Dôvera Case

The latest phase of case law development on the application of competition rules in the area of social solidarity-based insurance systems includes two key rulings of the CJEU: *Dôvera* and *Casa Regina Apostolorum.* ^55^ In the first of them, the CJEU assessed the nature of activities within the Slovak universal and compulsory health insurance system. The system operated based on a pluralistic model, in which the insured could freely choose between three health insurance companies — one state-owned (VšZP) and two private ones (Dôvera and Union). Insurance activity was heavily regulated. It could be conducted by public as well as private entities in the form of a regular joint stock company governed by private law. These companies not only competed with each other but also operated for profit and actually made profits.

The General Court annulled the Commission’s decision, asserting that the presence of competition among insurers and their ability to generate and distribute profits rendered their activities economic in nature, thus classifying them as undertakings subject to state aid rules.^56^ However, the GC ruling was overturned on appeal by the ECJ.^57^ The Court decided that the insurers were not undertakings within the meaning of EU competition law. The Court first reminded that “the EU law does not, in principle, detract from the powers of the Member States to organize their social security systems.”^58^ Following that, the Court held that according to the case law of the CJEU, an overall assessment of the scheme at issue was necessary, in which the following considerations had to be taken: “the pursuit, by the scheme, of a social objective, its application of the principle of solidarity, whether the activity carried out is non-profit-making, and State supervision of that activity.”^59^

The Court held that the Slovakian insurers operated under the principle of solidarity which was confirmed by the “compulsory nature of affiliation both for insured persons and for the insurance bodies; contributions which are fixed by law in proportion to the income of the insured persons and not the risk they represent individually on account of their age or state of health; the rule that compulsory benefits set by law are identical for all insured persons and do not depend on the amount of the contributions paid by each; and a mechanism for the equalisation of costs and risks through which schemes that are in surplus contribute to the financing of those with structural financial difficulties.”^60^

The ability of the insurance bodies managing the Slovak compulsory health insurance scheme to seek to make a profit was considered irrelevant since the status of an undertaking does not depend “on the legal status of the entity concerned but on all of the elements characterizing its activity.”^61^ Similarly, due to regulatory limitations, the possibility of using and distributing profits was considered not “liable to affect the social and solidarity character that arises from the actual nature of the activities concerned.”^62^

#### The Casa Regina Apostolorum Case

In *Casa Regina Apostolorum*, the sector-specific test was employed to assess the nature of the activities carried out by Italian hospitals operating within the framework of the national health insurance system. As such, the case concerned entities functioning at a downstream level relative to the health insurance funds examined in *Dôvera.* ^63^

The Italian healthcare system was financed directly by the social security contributions of the members and by state resources paid to a national sickness fund (SSN). The management of the SSN was mainly carried out on a regional level. The fund was financing the medical services provided in the framework of the system by public bodies and private providers under agreements. Thus, the services offered to patients were practically free of charge. The reforms of the Italian healthcare system which started in the 1990s gradually transformed public health entities (including hospitals) into companies subject to competition with public and private entities for care under the SSN and care provided at the individual request of patients.

Since healthcare services provided in the Lazio region were underfunded, the local government decided to reimburse the deficits of local public hospitals. In its decision, the Commission held that these payments were not state aid, since they were granted to entities of a noneconomic nature. The Commission clarified that the reforms of the Italian healthcare system, including changes to a model based on agreements with providers and free choice of a provider, did not change the core principles on which the system had been based, i.e., solidarity and universality of coverage.^64^ Similarly, the possibility of providing medical services by private practice doctors in hospitals did not call into question the noneconomic nature of the SSN.^65^

The General Court, citing the *Dôvera* case, upheld the Commission’s decision.^66^ The Court held that the Italian system fulfilled the criteria under which healthcare systems are considered noneconomic and explained that the introduction of a competitive element to the Italian system was merely intended to encourage operators to carry out their activity in accordance with the principles of sound management and did not alter the nature of that scheme.^67^ The Court made it clear that the reasoning that the principles of solidarity and universality preclude all competition and good management is incorrect.^68^ The Court confirmed the Commission’s view that the possibility of providing medical services outside the SSN by the entities operating under the system could not affect the system’s classification as having a social objective. Such activity should have been treated separately and assessed as economic.^69^ The General Court judgment was upheld on appeal, in its entirety by the Court of Justice.^70^

#### The Outcomes of the Third Phase of the Case Law Development

The judgments in *Dôvera* and *Casa Regina Apostolorum* appear to mark the culmination of the development of the sector-specific test for identifying undertakings. The first of these judgments extended the applicability of the sector-specific (concrete) test to the activities of entities managing health insurance schemes based on the principle of social solidarity, even where such activities were conducted for profit and under competitive conditions. The second judgment unequivocally affirmed that the sector-specific (concrete) test also applies to healthcare service providers, including those operating in a competitive environment.

As clearly emphasized by the Court in the *Dôvera* case, the sector-specific test is grounded in the characteristics of the healthcare system within which a given entity operates. The decisive criterion for excluding an entity from the category of “undertaking” is thus its operation within a healthcare system organized according to the principles of social solidarity — irrespective of whether the entity competes with other operators or even whether it pursues profit.

While, according to well-established case law, the nonprofit nature of an activity is not in itself sufficient to exclude its economic character,^71^ the pursuit of profit and the assumption of economic risk have long been regarded as key indicators of economic activity.^72^ However, in the context of the sectoral test, entities that operate on a for-profit basis and generate profit — thereby ostensibly adhering to market logic — may nonetheless not be classified as undertakings, provided that they operate within a system founded on solidarity-based principles.

The application of the general test of an undertaking — whether in its comparative or market-oriented formulation — to the activities of the Slovak insurance funds examined by the CJEU in the *Dôvera* case would likely have yielded a different outcome. The sector-specific (concrete) test adopted in *Dôvera* thus emerges not merely as a variant of the general undertaking test, but as a distinct and autonomous analytical framework. This development indicates the coexistence of two separate and mutually irreconcilable concepts of economic activity within the framework of EU competition law. Such a dualistic approach lacks justification in the Treaty provisions, which adopt a single, uniform definition of the undertaking and do not distinguish between entities based on the sector in which they operate.

This also implies that in the absence of any formal amendment to the Treaties — and thus, effectively through a judicial reinterpretation — the Court introduced a sector-specific exemption from the application of EU competition law. This exemption applies to activities carried out within the framework of general health or social insurance systems organized according to the principle of social solidarity.

Although under TFEU Article 6 healthcare is only a supportive competence of the Union and TFEU Article 168(7) recognizes the responsibilities of the Member States for the definition of their health policy and for the organization and delivery of health services and medical care, the Treaty, has not introduced the dedicated sector-based derogation from the rules of competition addressed to the healthcare sector.

This approach diverges from the logic traditionally underpinning the general concept of an undertaking, which holds that competition law is applicable whenever an entity engages in activities under competitive conditions — namely, in any context where the competitive process may be distorted or impaired. In contemporary healthcare systems, the functioning of competitive mechanisms can be undermined not only by Member States’ subsidies but also by anticompetitive conduct on the part of service providers.

As can be inferred from the reasoning in recent CJEU judgments — most notably in *Dôvera* — the Court’s stance appears to be guided primarily by pragmatic considerations. Specifically, the Court sought to allow Member States to implement and preserve efficiency-enhancing competition-based mechanisms within their universal healthcare systems, without subjecting those systems to the full rigor of EU competition law. This reflects deference to Member State competences in the field of healthcare, as recognized under TFEU Article 168(7).

These arguments are not entirely persuasive, however. Competition mechanisms can be effectively introduced by Member States without establishing a sectoral test of economic activity under the derogation from TFEU Article 106(2). Entities such as sickness funds and healthcare providers, even when fulfilling essential social functions and operating under obligations to provide services of general interest, may qualify for exemptions from the application of competition rules under this provision. Both medical services and the activities of sickness funds have, in fact, been recognized as potentially falling within the scope of this derogation.^73^ Moreover, the Commission’s SGEI Decision provides for a block exemption from the notification requirement under TFEU Article 108(3) for state aid granted as compensation for the provision of services of general economic interest by hospitals offering medical care, including, where applicable, emergency services.^74^

The *Dôvera* and *Casa Regina Apostolorum* rulings, however, resulted in a narrowing of the scope of application of the derogation under TFEU Article 106(2) by excluding from its ambit activities carried out within public healthcare systems based on the principle of social solidarity. If such activities are now deemed noneconomic and, consequently, TFEU Article 106(2) no longer applies to them, the question arises: to what kind of healthcare-related activities can this provision still be applied? It appears that its relevance is in general now limited to entities financed through private funding within the framework of voluntary insurance schemes. The difficulty, however, lies in the fact that such schemes offer limited scope for imposing public service obligations necessary to qualify as services of general economic interest.

One of the few post-*Dôvera* and *Casa Regina Apostolorum* scenarios in which TFEU Article 106(2) could still apply to healthcare activities is exemplified by the General Court’s judgment in *BUPA.* ^75^ In that case, payments made under a risk equalization scheme introduced by Irish legislation were considered compensation for the costs incurred by voluntary health insurers in discharging an SGEI entrusted to them. It should be noted, however, that mechanisms of this kind are not typical features of voluntary private insurance systems. In Ireland, their existence rather reflects the historical development of the private insurance market and its particularly significant social role — stemming, arguably, from the paid nature of healthcare services provided under the public system.

By contrast, the *IRIS* hospital network case — where the Commission concluded that payments made to public hospitals in Brussels qualified for the exemption under TFEU Article 106(2)— would likely be adjudicated differently under the current legal framework.^76^ Given the prevailing interpretation of activities carried out within public healthcare systems based on the principle of social solidarity, such hospitals would now be regarded as operating outside the scope of EU competition rules due to the noneconomic nature of their functions.

The *Dôvera* and *Casa Regina Apostolorum* rulings thus not only blurred the division line between noneconomic services and SGEIs but marginalized the role of the latter in the healthcare sector. It is challenging to identify any distinguishing feature, aside from sectoral membership, that differentiates services such as those evaluated by the CJEU in the *Dôvera* and *Casa Regina Apostolorum* cases from SGEIs in other sectors. Fundamentally, all SGEIs are entities entrusted with special tasks in the general interest. They typically operate based on principles of social solidarity and may compete with private operators, either within a market or for the market.

## The Consequences of the Application of the Sector-Specific Test to the Polish Healthcare System

### The Characteristics of the Polish Healthcare System

Article 68(1) of the Constitution of the Republic of Poland (1997) guarantees the right to health care for all individuals. Paragraph 2 of the same provision imposes a duty on public authorities to ensure equal access to publicly funded healthcare services, irrespective of a person’s financial situation.^77^

The structure of the Polish healthcare system is of a mixed nature and resembles the Italian model assessed by the CJEU in the *Casa Regina Apostolorum* case. Its foundation is a universal, compulsory health insurance scheme, primarily financed through contributions from the insured population, with employees forming the system’s financial backbone. A 9% contribution is deducted from employees’ salaries and subsequently transferred to the National Health Fund (*Narodowy Fundusz Zdrowia*, NFZ), which serves as the sole national sickness fund.^78^ For other insured groups — including students, the unemployed, and refugees — healthcare contributions are covered by the state budget.^79^ Medical services are generally provided free of charge, while the cost of outpatient pharmaceuticals is typically co-financed by the NFZ.

Healthcare services in Poland are delivered by entities of diverse legal status. A substantial portion of these is made up of Independent Public Healthcare Institutions (*Samodzielne Publiczne Zakłady Opieki Zdrowotnej*, SPZOZ), established by central or local public authorities, as well as by public universities.^80^ These institutions operate on a nonprofit basis,^81^ primarily delivering services financed by the NFZ. While they may offer services outside the general health insurance scheme, such services may only be provided to insured patients if the corresponding service is not covered by the NFZ. SPZOZ entities are thus characterized by their public character and adherence to nonprofit principles.

In addition to SPZOZ institutions, healthcare services may also be delivered by other public or private nonprofit entities, including research institutes, associations, foundations, governmental units at various levels, and religious organizations.^82^ Furthermore, the provision of healthcare services is open to private entrepreneurs within the meaning of Polish law — that is, to any natural person, legal person, or organizational unit without legal personality that has been granted legal capacity by a separate legal act, which conducts economic activity.^83^ Economic activity is defined as a profit-oriented, organized, and continuous operation conducted on one’s behalf.^84^ This category includes sole traders, partnerships, and companies, whether privately or publicly owned. These entities are permitted to operate both within the publicly funded healthcare system, under contracts with the NFZ, and outside of it, offering services for remuneration under market conditions.

Local authorities, particularly communes (*gminy*), occupy a central role within the Polish public healthcare system. Their statutory responsibility encompasses meeting the collective health-related needs of the local community, specifically by ensuring access to primary healthcare services.^85^ At the county (*powiat*) level, authorities are tasked with the oversight of general hospitals,^86^ whereas voivodeship (*województwo*) governments are responsible for specialist and psychiatric healthcare facilities.^87^ To fulfill these obligations, local governments are authorized to establish Independent Public Healthcare Institutions or other nonprofit medical entities, as well as profit-oriented companies. However, they are not required to directly provide healthcare services or to provide them through their own institutions if an adequate level of service from alternative providers is available within their jurisdiction.

The NFZ enters into contracts with service providers through a competitive selection process (tenders) or negotiations.^88^ It is obliged to ensure equal treatment and the promotion of fair competition throughout these proceedings.^89^

As a default procedure, tenders are conducted following specific evaluation criteria, including (1) quality of services; (2) comprehensiveness; (3) accessibility; (4) continuity of care; and (5) price.^90^ This procedure closely resembles the open procedure outlined in Article 27 of Directive 2014/24/EU on public procurement.^91^

Negotiated procedures may be employed only under exceptional circumstances expressly provided for by law. These include situations in which:

1. A prior tender has been declared invalid, and the terms of the new procedure remain unchanged.
2. There is an urgent need to conclude a healthcare services contract that could not have been anticipated.
3. The number of potential service providers capable of fulfilling the contract does not exceed five.^92^

The negotiated procedure is defined as a process for concluding contracts for the provision of healthcare services, whereby negotiations are conducted to determine the price, quantity, and conditions under which the services are to be provided. This procedure is carried out with a number of service providers sufficient to ensure the selection of the most advantageous offer, or multiple offers, while also maintaining procedural efficiency. As a rule, the number of participants should not be fewer than three, unless the specialized nature of the healthcare services or their limited availability justifies a smaller pool of qualified providers. Contracts are awarded to providers offering the most advantageous terms, irrespective of their legal status or organizational form.^93^

As indicated above, both public and private healthcare providers are eligible to participate in competitive tendering and negotiated procedures. Through their participation, they engage in competition for access to publicly financed contracts, effectively operating within a healthcare services market.

Separate regulatory provisions apply to so-called hospital networks — entities designated by the NFZ to ensure the delivery of specified hospital services.^94^ The list of participating hospitals is established every four years and includes entities that offer a statutory range of medical services.^95^ Both public and private providers may be included in the network; however, only hospitals operated by research institutes, medical universities, or the state may be designated as nationwide hospitals (i.e., those providing the most specialized, highest-level care).^96^ Inclusion in the hospital network guarantees financing from the NFZ without requiring participation in competitive tenders or negotiated procedures.

Parallel to the public system, Poland has developed a robust private healthcare market, in which services are financed directly by patients or through private health insurance schemes. Although private medical activity is subject to regulatory oversight, it is conducted on competitive market terms. Providers have discretion in setting service prices, and private insurers typically enjoy broad autonomy in assessing insurance risk, including the right to deny coverage in cases of high-risk applicants.

### Distortions of Competition Resulting from the Sector-Specific Test of the Undertaking

In light of the *FENIN* ruling and subsequent case law of the CJEU, it is well established that the NFZ, as a public sickness fund operating within a system based on the principles of social solidarity and lacking competition, is not considered an undertaking within the meaning of EU competition law. This conclusion would remain unchanged even under the application of the general undertaking test. Likewise, applying the sector-specific test to activities conducted within the public healthcare system leads to the same finding: such activities, by virtue of their systemic organization and solidarity-based principles, do not constitute economic activities, regardless of whether the entities performing them operate on a for-profit or nonprofit basis.

However, the outcome would differ considerably if the market-based approach — either in its comparative or market (competition) variant — were applied. Healthcare providers operating under the public health insurance scheme in Poland do, in fact, compete across multiple dimensions. First, they participate in competitive procedures organized by the NFZ, where both public and private providers — irrespective of legal form or profit orientation — compete for contracts. Offers are evaluated based on standard competition parameters such as price and quality. Second, even within the scope of the negotiated (restricted) procedure, competition exists: at least three providers are required to ensure the selection of the most advantageous offer, once again based on price and service conditions. Third, albeit to a lesser extent, healthcare entities compete for inclusion on the NFZ’s list of designated hospitals. While this decision is made based primarily on the scope of services provided, the process still involves elements of competition.

In at least the first two cases, healthcare providers compete intensely for access to public financing, which serves as a proxy for market access. Many of these providers operate on a for-profit basis, and their market position is not fundamentally different from undertakings entrusted with the SGEIs — for instance, in the local public transport sector — where entities similarly compete in public procurement procedures. Paradoxically, these healthcare providers often face more intense competition than transport providers, which in Poland may benefit from *in-house* awards exempt from procurement rules, a possibility not available in the healthcare sector under current NFZ contracting rules.

The exclusion of entities competing for NFZ contracts from the definition of an undertaking under EU competition law raises serious concerns regarding competitive neutrality. One major distortion arises from the privileged access of public providers — those established or controlled by state or local authorities — to public financing. These entities can receive funding from their public founders or shareholders without being subject to state aid rules or the requirements of national legislation transposing the EU Transparency Directive. As a result, they are often able to leverage this advantage in NFZ tender procedures, creating barriers to entry and expansion for private providers. This advantage also extends to the hospital network list. Public entities, through easier access to capital, may be better positioned to expand their range of services, thereby increasing their eligibility for inclusion in the list maintained by the NFZ.

A second, and no less significant, concern arising from the exclusion of activities within the public healthcare system from the scope of EU competition rules is the potential for anticompetitive practices by entities operating within this framework — particularly in the form of cartels. Where such activities fall outside the ambit of competition law, service providers may engage in collusive practices, such as group boycotts or bid rigging, aimed at eliminating more efficient competitors from the market. This risk is not merely theoretical. A notable example from Polish enforcement practice illustrates this point. In 2012, the Polish Competition Authority found that nine public hospitals in Lublin had infringed the prohibition on anticompetitive agreements. These hospitals had failed to secure contracts in an NFZ tender for the provision of specialist diagnostic services, losing to private providers with whom they maintained subcontracting arrangements.^97^ In response, they collectively terminated these agreements, thereby preventing the private providers from fulfilling their contracts with the NFZ.

If such conduct were assessed under the sector-specific test, it would arguably fall outside the scope of EU competition law, as the conduct occurred entirely within the framework of the public healthcare system. Although such behavior could still be sanctioned under national competition law, the applicability of these rules would be contingent upon the absence of any effect on trade between Member States. In cases where such an effect exists, the convergence rule under Article 3(2) of Regulation 1/2003 would preclude the application of stricter national provisions, effectively leaving a regulatory gap.^98^

The exclusion of entities operating within the Polish public health insurance system from the scope of EU state aid rules and the concept of undertaking under competition law poses a broader challenge to the integrity and functioning of competitive mechanisms. These mechanisms are foundational to the structure of the Polish healthcare system, particularly in relation to service contracting. The challenges outlined above could be mitigated by recognizing entities operating within this system as undertakings entrusted with the provision of SGEIs. Such an approach would ensure oversight over distortive state aid practices and deter anticompetitive conduct, without obstructing the continued public financing of healthcare services. Importantly, this would enable compensation for the provision of SGEIs, thereby facilitating a level playing field among all entities participating in the Polish healthcare system — whether public or private, for-profit or nonprofit.

## Conclusions

The development of the CJEU’s case law has resulted in the emergence of two distinct and mutually incompatible concepts of economic activity within the framework of EU competition law. The third phase of this jurisprudential evolution, encompassing the *Dôvera* and *Casa Regina Apostolorum* judgments, has extended the scope of application of the sector-specific (or concrete) test initially formulated in *Poucet.* The application of this test has led to the exclusion from EU competition rules of activities conducted within public healthcare systems organized according to the principle of social solidarity — even when such activities are performed under competitive conditions and for profit. In contrast, applying the general (or abstract) test of economic activity would lead to the opposite conclusion. This divergence in approach is not explicitly supported by the provisions of the Treaty and raises serious concerns regarding the effectiveness of competition mechanisms introduced by Member States within their healthcare systems. Moreover, this jurisprudential shift has blurred the conceptual boundary between noneconomic services and services of general economic interest, effectively marginalizing the role of the latter in the healthcare sector.

In the context of the Polish healthcare system, the exclusion of activities within the public healthcare framework from the scope of competition law risks conferring an undue market advantage on public providers. These entities are often able to supplement NFZ reimbursements with additional subsidies from local authorities or other public bodies granted outside of the state aid control. Furthermore, healthcare providers operating within the public system, having been consistently excluded from competition law oversight, may engage in anticompetitive practices without regulatory consequences, provided such conduct does not affect trade between Member States. This situation calls into question the effectiveness of competition-based mechanisms, including the use of public tenders by the National Health Fund, which remains the primary instrument for contracting healthcare services.

## References

[1] V. Lourie, “‘Undertaking’ as a Jurisdictional Element for the Application of EC Competition Rules,” Legal Issues of Economic Integration 29, no. 2 (2002): 143–176.

[2] *Mannesmann AG v High Authority of the European Coal and Steel Community*, case 19/61, 1962 E.C.R. 357, at 371.

[3] *Höfner and Elser v. Macrotron*, case C-40/91, 1991 E.C.R. I-01979.

[4] Id., at para. 21; see also *HFB Holding für Fernwärmetechnik Beteiligungsgesellschaft mbH & Co. KG and Others v Commission*, case T-9/99, 2002 E.C.R. II-01487, at para. 54, where the Court held that “Article [101 (1) of the Treaty is aimed at economic units which consist of a unitary organisation of personal, tangible and intangible elements, which pursue a specific economic aim on a long-term basis and can contribute to the commission of an infringement of the kind referred to in that provision”; see also the case law cited therein: *Shell v Commission*, case T-11/89, 1992 E.C.R. II-757, at para. 311, and *Mo Och Domsjö v Commission*, case T-352/94, 1998 E.C.R. II-1989, at para. 87.

[5] *Federación Española de Empresas de Tecnología Sanitaria (FENIN) v Commission*, case C-205/03 P, 2006 E.C.R. II-6295 [hereinafter cited as *FENIN*], Opinion of AG Poiares Maduro, at paras. 11–15.

[6] See *Höfner and Elser v. Macrotron*, *supra* note 3; *Ambulanz Glöckner, Landkreis Südwestpfalz and others*, case C-475/99, 2001 E.C.R. I-9089 [hereinafter cited as *Ambulanz Glöckner*].

[7] *Commission v Italy*, case 118/85, 1987 E.C.R. 2599, at paras. 3 and 7; *Commission v Italy*, case C-35/96, 1998 E.C.R. I-3851, at para. 36; *Pavel Pavlov et al. v Stichting Pensioenfonds Medische Specialisten*, joined cases C-180-184/98, 2000 E.C.R. I-6451 [hereinafter cited as *Pavel Pavlov*]; *Cisal di Battistelo Venanzio*, case C-218/00, 2002 E.C.R., s. I-691, at paras. 22 and 23; see *Ambulanz Glöckner*, *supra* note 6, at para. 19.

[8] See *FENIN*, *supra* note 5, at para. 13.

[9] Similarly, M. Kleis and P. Nicolaides, “The Concept of Undertaking in Education and Public Health Systems,” European State Aid Law Quarterly 5, no. 3 (2006): 505–508, at 506, 10.21552/ESTAL/2006/3/83.

[10] See *Commission v Italy*, Case 118/85, *supra* note 7, at para. 7; see *Pavel Pavlov*, *supra* note 7, at para. 75; see *Ambulanz Glöckner*, *supra* note 6, at para. 19; *Wouters et al.*, case C-309/99, 2002 E.C.R. I-1577, at para. 47; *Aeroports de Paris*, case C-82/01 P, 2002 E.C.R. I-9297, at para. 79; see *Commission v Italy*, case C-35/96, *supra* note 7, at para. 36; see *Cisal di Battistelo Venanzio*, *supra* note 7, at paras. 22 and 23.

[11] *Ministero dell’Economia e delle Finanze v Cassa di Risparmio di Firenze SpA and Fondazione Cassa di Risparmio di San Miniato and Cassa di Risparmio di San Miniato SpA*, case C-222/04, 2006 E.C.R. I-289 [hereinafter cited as *Ministero dell’Economia e delle Finanze*]; Tax measures for banking foundations implemented by Italy, Commission Decision 2003/147/EC, C 54/2000/EC (ex NN 70/2000), 2003 OJ (L 55), at paras. 42–47; see also A. Biondi, “Judgment in Case C-222/04 Ministero dell’Economia e delle Finanze v. Cassa di Risparmio di Firenze SpA et al.,” European Staid Aid Law Quarterly 5, no. 2 (2006): 371–376.

[12] *SAT Fluggesellschaft (Eurocontrol)*, case C-364/92, 1994 E.C.R. I-43, at para. 30; see also *Wouters at al.*, *supra* note 10, at para. 57, and *Motosykletistiki Omospondia Ellados NPID (MOTOE) v Elliniko Dimosio*, case C-49/07, 2008 E.C.R. I-4863, at para. 24.

[13] *Diego Cali & Figli Srl v Servizi ecologici porto di Genova SpA (SEPG)*, case C-343/95, 1997 E.C.R. I-1547 [hereinafter cited as *Cali & Figli*], at para. 22.

[14] See Commission Notice on the Notion of State Aid as referred to in Article 107(1) of the Treaty on the Functioning of the European Union, 2016 OJ (C 262/1), at para. 17, and the CJEU case law and Commission Decisions cited therein, in the following cases: *Ministero dell’Economia e delle Finanze*, *supra* note 11, at paras. 107–118, and 125; *Commission v Italy*, case 118/85, *supra* note 7, at paras. 7 and 8; *Corinne Bodson v SA Pompes funèbres des régions libérées*, case 30/87, 1988 E.C.R. 2479, at para. 18; *SAT/Eurocontrol*, *supra* note 12, at paras. 27 and 30; Calì & Figli, supra note 13, at paras. 20, 22, and 23; United Kingdom — Aid to Forensic Science Services, case SA.32820 (2011/NN), Commission Decision, 2012 OJ (C 29/4), at para. 8; *Selex Sistemi Integrati v Commission*, case C-113/07 P, 2009 E.C.R. I-02207, at para. 71; Belgium — Aid to port authorities, Commission Decision, case N 438/02, 2002 OJ (C 284/2); Lithuania — Allotment of subsidies to the State Enterprises at the Correction Houses, Commission Decision, case N 140/06, 2006 OJ (C 244/12); Germany — GRW land development scheme for industrial and commercial use, Commission Decision, case SA.36346, 2014 OJ (C 141/1); *Compass-Datenbank GmbH*, case C-138/11, ECLI:EU:C:2012:449, at para. 40.

[15] *Christian Poucet v Assurances Générales de France and Caisse Mutuelle Régionale du Languedoc-Roussillon*, joined cases C-159/91 and C-160/91, 1993 E.C.R. I-637 [hereinafter cited as *Christian Poucet v Assurances Générales de France*].

[16] Id., at paras. 6 and 10.

[17] See *Christian Poucet v Assurances Générales de France*, *supra* note 15, at para. 18.

[18] J. L. Buendia Sierra, Exclusive Rights and State Monopolies under EC Law (Oxford University Press, 1999): at 52; S. Belhaj and J. van de Gronden, “Some Room for Competition Does not Make a Sickness Fund an Undertaking. Is EC Competition Law Applicable to the Health Care Sector? (Joined Cases C-264/01, C-306/01, C-453/01 And C-355/01 AOK),” *European Competition Law Review* 25, no. 11 (2004): 682–687.

[19] A. Winterstein, “Nailing the Jellyfish: Social Security and Competition Law,” European Competition Law Review 20 (1999): 324–333, at 333.

[20] N. Boeger, “Solidarity and EC Competition Law,” European Law Review 32, no. 3 (2007): 319–340.

[21] C. Barnard, ** *“* **EU Citizenship and the Principle of Solidarity,” in Social Welfare and the Law, ed. M. Dougan and E. Spaventa (Bloomsbury, 2005): 165.

[22] See *Cisal di Battistelo Venanzio*, *supra* note 7.

[23] See *Cisal di Battistelo Venanzio*, *supra* note 7, at paras. 34 and 37.

[24] See *Cisal di Battistelo Venanzio*, *supra* note 7, at para. 42.

[25] *Fédération Française des Sociétés d’Assurance, Société Paternelle-Vie, Union des Assurances de Paris-Vie and Caisse d’Assurance et de Prévoyance Mutuelle des Agriculteurs* v *Ministère de l’Agriculture et de la Pêche*, case C-244/94, 1995 E.C.R. I-4013 [hereinafter cited as *Fédération Française des Sociétés d’Assurance*].

[26] *Albany International BV v Stichting Bedrijfspensioenfonds Textielindustrie*, case C-67/96, 1999 E.C.R. I-5751.

[27] *Brentjens’ Handelsonderneming BV v Stichting Bedrijfspensioenfonds voor de Handel in Bouwmaterialen*, joined cases C-115/97 to C-117/97, 1999 E.C.R. I-6025.

[28] See *Fédération Française des Sociétés d’Assurance*, *supra* note 25, at para. 21.

[29] Notably, the amount of the premium paid by the insured was not determined by the level of individual risk incurred. See *Fédération Française des Sociétés d’Assurance*, *supra* note 25, at para. 17.

[30] See *Albany International BV v Stichting Bedrijfspensioenfonds Textielindustrie*, *supra* note 26, at para. 84.

[31] See *Albany International BV v Stichting Bedrijfspensioenfonds Textielindustrie*, *supra* note 26, at paras. 86 and 123.

[32] J.W. van de Gronden “Purchasing care: economic activity or service of general (economic) interest?” European Competition Law Review 25 (2004): 84–86, at 87

[33] Id. See also: J.B. Cruz, “Beyond Competition: Services of General Interest and EC Law,” in EU Law and the Welfare State, ed. G. de Burca (Oxford University Press, 2005): 184, 10.1093/acprof:oso/9780199287413.001.0001; J.W. van de Gronden “Services of general interest and the concept of undertaking: does EU competition law apply?” *World Competition* 41, no. 2 (2018): 197–223, at 197; J.W. van de Gronden and M. Guy, “The role of EU competition law in health care and the ‘undertaking’ concept,” *Health Economics, Policy and Law* 16 (2021): 76–89, at 79. 32349857

[34] *AOK Bundesverband et al.*, joined cases C-264/01, C-306/01, C-354/01, and C-355/01, 2004 E.C.R. I-2493.

[35] Id., at para. 54.

[36] See *AOK Bundesverband et al.*, supra note 34, at para. 56.

[37] See *AOK Bundesverband et al.*, supra note 34, at para. 54.

[38] See Belhaj and van de Gronden, *supra* note 18, at 684–685.

[39] See Belhaj and van de Gronden, *supra* note 18, at 685.

[40] See Cruz, *supra* note 33, at 184.

[41] See Cruz, *supra* note 33, at 184.

[42] M. Krajewski and M. Farley, “Non-Economic Activities in Upstream and Downstream Markets and the Scope of Competition Law After FENIN,” European Law Review 32, no. 1 (2007): 111–124; see also T. Prosser, *The Limits of Competition Law. Public Service and EC Law: Markets and Public Services* (Oxford University Press, 2005): 130; similarly, see Cruz, *supra* note 33, at 184.

[43] The Court of First Instance, renamed the General Court of the EU after the Treaty of Lisbon of 2007, is a lower instance court of the Court of Justice of the EU.

[44] *Federación Nacional de Empresas de Instrumentación Científica, Médica, Técnica y Dental (FENIN) v Commission*, case T-319/99, 2003 E.C.R. II-357.

[45] P.J. Slot, “Applying the Competition Rules in the Healthcare Sector,” European Competition Law Review 24, no. 11 (2003): 580–593, at 587; V. Louri, “The FENIN Judgment: The Notion of Undertaking and Purchasing Activity,” *Legal Issues of Economic Integration* 32, no. 1 (2005): 87–97, at 89; see Krajewski and Farley, *supra* note 42, at 117.

[46] See *FENIN*, *supra* note 5, at para. 13.

[47] Krajewski and Farley, *supra* note 42, at 123.

[48] See *Federación Nacional de Empresas de Instrumentación Científica*, *supra* note 44, at para. 40.

[49] See *Pavel Pavlov*, *supra* note 7.

[50] See van de Gronden and Guy, *supra* note 33, at 76, 81–82.

[51] Commission Notice on the Notion of State Aid as referred to in Article 107(1) of the Treaty on the Functioning of the European Union, 2016 OJ (C 262/1).

[52] See *Wyjaśnienia i pomocne pliki (Explanations and Helpful Files)* (Polish Competition Authority (UOKiK)), https://uokik.gov.pl/wyjasnienia-wzory-oraz-pomocne-pliki, (last visited April 22, 2025).

[53] Id.

[54] See *Wyjaśnienia i pomocne pliki*, *supra* note 52 .

[55] *Commission v Dôvera zdravotná poist’ovňa & Union zdravotná poist’ovňa*, cases C‑262/18 P and C‑271/18 P, ECLI:EU:C:2019:1144, and *Casa Regina Apostolorum della Pia Società delle Figlie di San Paolo v Comission*, case T‑223/18, ECLI:EU:T:2021:315 [hereinafter cited as *Casa Regina Apostolorum*].

[56] *Dôvera zdravotná poistʼovňa v Commission*case T‑216/15, ECLI:EU:T:2018:64, at paras. 62–66.

[57] See *Commission v Dôvera*, *supra* note 55.

[58] See *Commission v Dôvera*, *supra* note 55, at para. 30.

[59] See *Commission v Dôvera*, *supra* note 55, at para. 30, and the case law cited therein in the following cases: *Poucet and Pistre*, cases C‑159/91 and C‑160/91, EU:C:1993:63, at paras. 8–10, 14, 15, and 18; *Cisal di Battistelo Venanzio*, *supra* note 7, at paras. 34, 38, and 43; *AOK Bundesverband et al.*, *supra* note 34, at paras. 47–50; *Kattner Stahlbau*, case C‑350/07, EU:C:2009:127, at paras. 35, 38, and 43; *AG2R Prévoyance*, case C‑437/09, EU:C:2011:112, at paras. 43–46.

[60] See *Commission v Dôvera*, *supra* note 55, at para. 32.

[61] See *Commission v Dôvera*, *supra* note 55, at para. 39.

[62] See *Commission v Dôvera*, *supra* note 55, at para. 40.

[63] See *Casa Regina Apostolorum*, *supra* note 55; see also M. Guy, “On the Quiet Power of National Decisions: Hospitals, State Aid, and Services of General Economic Interest,” Public Health Markets and Law Symposium, Journal of Law, Medicine and Ethics 53, no. 3 (2025): xxx–xxx. 10.1017/jme.2025.10146 PMC12877730 40696818

[64] See *Casa Regina Apostolorum*, *supra* note 55, at paras. 27, 28, and 29.

[65] See *Casa Regina Apostolorum*, *supra* note 55, at para. 30.

[66] See *Casa Regina Apostolorum*, *supra* note 55, at paras. 2 and 34.

[67] See *Casa Regina Apostolorum*, *supra* note 55, at para. 163.

[68] See *Casa Regina Apostolorum*, *supra* note 55, at para. 176.

[69] See *Casa Regina Apostolorum*, *supra* note 55, at paras. 181, 182, and 183.

[70] *Casa Regina Apostolorum della Pia Società delle Figlie di San Paolo v Commission*, case C-492/21 P, 2023 ECLI:EU:C:2023:354.

[71] See inter alia: *Denmark v Commission*, case T‑364/20, ECLI:EU:T:2024:125, at para. 146; *Van Landewyck*, joined cases 209/78 to 215/78 and 218/78, ECLI:EU:C:1980:248, at para. 88; see *Fédération Française des Sociétés d’Assurance*, *supra* note 25, at para. 21; see *MOTOE*, *supra* note 12, at paras. 27 and 28; see *Ambulanz Glöckner*, *supra* note 6, at para. 19; *Havenbedrijf Antwerpen and Maatschappij van de Brugse Zeehaven v Commission*, T‑696/17, ECLI:EU:T:2019:652, at para. 46; *Mitteldeutsche Flughafen and Flughafen Leipzig-Halle v Commission*, case C‑288/11 P, ECLI:EU:C:2012:821, at para. 50; *Spain v Commission*, case T‑808/14, ECLI:EU:T:2016:734, at para. 55.

[72] *Pavlov and Others*, joined cases C-180/98 to C-184/98, ECLI:EU:C:2000:428, at paras. 75 and 77.

[73] See *British United Provident Association Ltd (BUPA) and Others v Commission*, case T-289/03, 2008 E.C.R. II-00081; Commission Decisions: State aid implemented by Belgium — Public financing of Brussels public IRIS hospitals, Commission Decision, case SA.19864 - 2014/C (ex 2009/NN54), 2016 OJ (L 351/68); State aid — Ireland Risk Equalisation Scheme for 2013, Commission Decision, case SA.34515 (2013/NN), 2013 OJ (C 204/2); State aid — Ireland Risk Equalisation Scheme Commission Decision, case SA.41702 (2016/NN), 2016 OJ (C 104/1); State aid — Ireland Prolongation of the Risk Equalisation Scheme Commission Decision, case SA.58851, 2022 OJ (C 17/1); State aid — Ireland Risk Equalisation Scheme 2022, Commission Decision, case SA.64337, 2022 OJ (C 196/4).

[74] Commission Decision of 20 December on the application of Article 106(2) of the Treaty on the Functioning of the European Union to State aid in the form of public service compensation granted to certain undertakings entrusted with the operation of services of general economic interest, 2012 OJ (L 7/3), at Art. 2 (1) (d).

[75] *British United Provident Association Ltd (BUPA) and Others v Commission*, case T-289/03, 2008 OJ (C 79/25).

[76] State aid implemented by Belgium — Public financing of Brussels public IRIS hospitals (case SA.19864 — 2014/C (ex 2009/NN54) Commission Decision 2016/2327, 2016, OJ (L 351/68).

[77] The Constitution of the Republic of Poland as adopted by the National Assembly on 2nd April 1997, Journal of Laws 1997 No. 78 item 483 as amended.

[78] Act of 27 August 2004 on Healthcare Services Financed from Public Funds, Journal of Laws 2024 No.146 consolidated text as amended [hereinafter cited as Act of 27 August 2004, Healthcare Services], at Art. 79 (1).

[79] Id., at Art. 94.

[80] Act of 15 April 2011 on Healthcare Activity, Journal of Laws 2025 No.450 consolidated text as amended, at Art. 50a (1).

[81] Id., at Art. 57.

[82] See Act of 15 April 2011 on Healthcare Activity, *supra* note 80, at Art. 4.

[83] See Act of 15 April 2011 on Healthcare Activity, at Art. 4; Act of 6 March 2018 on Entrepreneurs, Journal of Laws 2024 No. 236 consolidated text as amended, at Art. 4(1).

[84] See Act of 6 March 2018 on Entrepreneurs, *supra* note 83, at Art. 3.

[85] Act of 8 March 1990 on Municipal Self-Government, Journal of Laws 2024 No. 1465 consolidated text as amended, at Art. 7(1)(5).

[86] Act of 5 June 1998 on County Self-Government, Journal of Laws 2024 No. 107 consolidated text as amended, at Art. 4(1)(2).

[87] Act of 5 June 1998 on the Voivodeship Self-Government, Journal of Laws 2024 No. 566 consolidated text as amended, at Art. 14(1)(2).

[88] See Act of 27 August 2004, Healthcare Services, *supra* note 78, at Art. 139 (1).

[89] See Act of 27 August 2004, Healthcare Services, *supra* note 78, at Art. 134 (1).

[90] See Act of 27 August 2004, Healthcare Services, *supra* note 78, at Art. 148 (1).

[91] The Directive of the European Parliament and of the Council 2014/24/EU of 26 February 2014 on public procurement and repealing Directive 2004/18/EC, 2014 OJ (L 94/65).

[92] See Act of 27 August 2004, Healthcare Services, *supra* note 78, at Art. 144.

[93] See Act of 27 August 2004, Healthcare Services, *supra* note 78, at Art. 143 (2).

[94] See Act of 27 August 2004, Healthcare Services, *supra* note 78, at Art. 95l-95n.

[95] See Act of 27 August 2004, Healthcare Services, *supra* note 78, at Art. 95m.

[96] See Act of 27 August 2004, Healthcare Services, *supra* note 78, at Art. 95m(3)(2).

[97] Decision of the President of the OCCP No. RLU-31/2012 of 18 December 2012.

[98] Council Regulation (EC) No 1/2003 of 16 December 2002 on the implementation of the rules on competition laid down in Articles 81 and 82 of the Treaty, 2003 OJ (L1/1).

